# Matching Quantitative MRI Parameters with Histological Features of Treatment-Naïve *IDH* Wild-Type Glioma

**DOI:** 10.3390/cancers13164060

**Published:** 2021-08-12

**Authors:** Gabriele D. Maurer, Julia Tichy, Patrick N. Harter, Ulrike Nöth, Lutz Weise, Johanna Quick-Weller, Ralf Deichmann, Joachim P. Steinbach, Oliver Bähr, Elke Hattingen

**Affiliations:** 1 Senckenberg Institute of Neurooncology, Goethe University Hospital, 60528 Frankfurt am Main, Germany; julia.tichy@gmx.de (J.T.); joachim.steinbach@kgu.de (J.P.S.); oliver.baehr@klinikum-ab-alz.de (O.B.); 2 Institute of Neurology (Edinger Institute), Goethe University Hospital, 60528 Frankfurt am Main, Germany; patrick.harter@kgu.de; 3 German Cancer Consortium (DKTK), Partner Site Frankfurt/Mainz, 60590 Frankfurt am Main, Germany; 4 German Cancer Research Center (DKFZ), 69120 Heidelberg, Germany; 5 Frankfurt Cancer Institute (FCI), 60596 Frankfurt am Main, Germany; 6 Brain Imaging Center, Goethe University, 60528 Frankfurt am Main, Germany; noeth@med.uni-frankfurt.de (U.N.); deichmann@med.uni-frankfurt.de (R.D.); 7 Division of Neurosurgery, Dalhousie University Halifax, Halifax, NS B3H 4R2, Canada; weise@dal.ca; 8 Department of Neurosurgery, Goethe University Hospital, 60528 Frankfurt am Main, Germany; johanna.quick@kgu.de; 9 Department of Neurology, Klinikum Aschaffenburg-Alzenau, 63739 Aschaffenburg, Germany; 10 Institute of Neuroradiology, Goethe University Hospital, 60528 Frankfurt am Main, Germany; elke.hattingen@kgu.de

**Keywords:** quantitative MRI, glioblastoma, image-guided tissue acquisition, hypoxia

## Abstract

**Simple Summary:**

MRI plays an important role in the classification of structural brain changes and quantitative MRI can provide more and different information than conventional MRI. A comparison with neuropathological parameters helps to expand the understanding of (patho)physiological concepts but has hardly been carried out so far. In this prospective study, we correlated quantitative MRI data with histological features of 25 patients with isocitrate dehydrogenase (*IDH*) wild-type glioma. We found that the relative shortening of T1 relaxation time after the application of contrast agent was pronounced in the presence of tumor cells and vascular proliferates, indicating a disruption of the blood brain barrier (BBB). T2′ relaxation time, depending on local deoxyhemoglobin concentration, correlated with the vessel density but not with immunopositivity for endogenous markers of hypoxia.

**Abstract:**

Quantitative MRI allows to probe tissue properties by measuring relaxation times and may thus detect subtle changes in tissue composition. In this work we analyzed different relaxation times (T1, T2, T2* and T2′) and histological features in 321 samples that were acquired from 25 patients with newly diagnosed *IDH* wild-type glioma. Quantitative relaxation times before intravenous application of gadolinium-based contrast agent (GBCA), T1 relaxation time after GBCA as well as the relative difference between T1 relaxation times pre-to-post GBCA (T1rel) were compared with histopathologic features such as the presence of tumor cells, cell and vessel density, endogenous markers for hypoxia and cell proliferation. Image-guided stereotactic biopsy allowed for the attribution of each tissue specimen to its corresponding position in the respective relaxation time map. Compared to normal tissue, T1 and T2 relaxation times and T1rel were prolonged in samples containing tumor cells. The presence of vascular proliferates was associated with higher T1rel values. Immunopositivity for lactate dehydrogenase A (LDHA) involved slightly longer T1 relaxation times. However, low T2′ values, suggesting high amounts of deoxyhemoglobin, were found in samples with elevated vessel densities, but not in samples with increased immunopositivity for LDHA. Taken together, some of our observations were consistent with previous findings but the correlation of quantitative MRI and histologic parameters did not confirm all our pathophysiology-based assumptions.

## 1. Introduction

MRI is crucial for diagnosis, therapy planning and monitoring of gliomas. At present, it is mainly used for determining morphological features, such as the tumor size, contrast enhancement, necrosis and its spatial relationship to functional brain structures. In addition to conventional MRI, techniques such as perfusion-weighted imaging, functional MRI, MR spectroscopy and positron emission tomography (PET) are frequently applied as they also provide valuable information about different pathophysiological aspects of the tumor and its microenvironment. Glioblastomas in particular exhibit a marked intratumor heterogeneity, posing the risk of biopsy sampling error. In contrast, imaging facilitates the non-invasive characterization of the entire tumor.

T1 indicates how fast the excited tissue protons emit the energy absorbed from the radio-frequency pulse and realign with the external magnetic field. T2 is the time constant of the decay process of transverse magnetization due to interactions between the magnetic fields of adjacent protons. The time constant T2* is influenced by several dephasing processes, such as magnetic field inhomogeneity, magnetic susceptibility, chemical shift and spin–spin interaction [1]. T2′ can be considered a version of T2* which is corrected for spin–spin interactions, being defined by 1/T2* = 1/T2 + 1/T2′. Both T2* and T2′ values sensitively reflect modifications in tissue composition, though T2′ better indicates the impact of blood oxygenation [2].

However, subtle tissue changes may be missed on conventional MRI upon visual assessment as they display mixed tissue contrasts, depending on hardware and protocol settings [3]. Quantitative MRI (qMRI) methods yield specific tissue parameters, e.g., the relaxation times T1, T2 and T2* in milliseconds (ms), thus enabling a more objective quantitative assessment of tissue properties. Significant deviations of parameters from normal control values reflect tissue alterations, facilitating the identification of brain pathologies [4]. For example, it has been demonstrated that synthetic data sets derived from qMRI maps may improve the visibility of brain structures such as the deep cerebellar nuclei [5] and pathologies such as brain tumors [6] or focal cortical dysplasia [7,8]. In glioblastoma, maps of quantitative T1-relaxation time differences pre-to-post intravenous application of a gadolinium-based contrast agent (GBCA) have been shown to better visualize GBCA leakage than conventional T1-weighted MRI, indicating tumor cell infiltration in and beyond the peritumoral edema [9,10]. Furthermore, in patients treated with bevacizumab, differential T1 and T2 mapping allowed for the detection of glioblastoma progression at an earlier stage than conventional MRI [11,12]. Despite such encouraging results, observations from qMRI have rarely been histopathologically verified, e.g., in nine patients with epilepsy surgical temporal lobe resection [13] and in 15 patients with resection of pancreatic cancer [14].

In this prospective study, treatment-naïve patients with suspected high-grade glioma underwent qMRI before tumor biopsies were taken. The goal of the study was to correlate MRI parameter values with biologic findings from samples taken via image-guided stereotactic biopsy, thus gaining insights into the relationship between tissue alterations and relaxation time changes. Of particular interest was to investigate whether changes in T2′, indicating altered oxygen extraction, correlated with indicators of hypoxia in tissue samples. In addition to routine diagnostics for the classification of the tumor, e.g., the determination of the isocitrate dehydrogenase (*IDH*) mutation status and the O6-methylguanine-DNA methyltransferase (*MGMT*) promoter methylation status, workup included the immunohistochemical detection and quantification of lactate dehydrogenase A (LDHA), carbonic anhydrase IX (CAIX) and Ki67, as well as the quantification of tumor cells, vessels and the extent of necrosis in hematoxylin-eosin (HE) stained tissue sections.

## 2. Patients, Materials and Methods

### 2.1. Patients

This prospective study was approved by the scientific board of the University Cancer Center Frankfurt and the local ethics committee (SNO 01-11). Patients were considered eligible if (i) they were able to give written informed consent, (ii) no contraindication for MRI and GBCA was present, (iii) a high-grade glioma was suspected on conventional MRI and (iv) the multidisciplinary tumor board recommended a stereotactic biopsy of the lesion. In view of the specific in-house experience [15] and the literature, we assumed that biopsy would confirm the diagnosis in 95% of the cases, that high-grade glioma would be present in 90% and that we would be able to obtain approximately 10 samples per patient. Using G*Power [16] and presuming an effect size d = 0.5, a significance level α = 0.05 and a power (1 − β) = 0.95, we aimed to analyze at least 210 glioma samples, i.e., 25 patients with evaluable qMRI. Thirty-four patients agreed to participate in the study. However, five of them finally opted for tumor resection and two waived biopsy and hereby histological confirmation of the diagnosis. In two patients, the biopsy revealed a metastasis of a squamous cell carcinoma and an adenocarcinoma, respectively. Basic information on the remaining 25 patients and their tumors is given in Table 1. The MRI scans were performed 1–3 days before biopsy. Twenty-three of these patients died from their disease. Two patients were transferred from our hospital to a hospice; we have no information on their survival. Part of these patients have also been included in a different study comparing signal differences between pre- and post GBCA in conventional and qMRI [9].

### 2.2. Magnetic Resonance Imaging and Processing

MRI was performed on a 3T whole body scanner (Magnetom Verio, Siemens, Erlangen, Germany), using a body transmit and an eight-channel phased-array head receive coil. Except for T1 post GBCA, all relaxation times were determined before the intravenous administration of GBCA. Sequence parameters for qMRI are indicated in Table 2. Supplementary Figure S1 illustrates the evaluation of T2, T2* and T2′ maps, showing data from one representative patient.

For quantitative T2-mapping, five turbo spin echo datasets with different echo times were acquired (in-plane resolution 1.25 mm isotropic, 25 slices, 2 mm thickness, no gap). In order to suppress k-space lines affected by motion, data for T2* mapping were acquired with 100%, 50% and 25% of k-space lines covering central k-space; then, motion correction was performed as described in the literature [19]. Furthermore, the sensitivity profile B1 of the transmit coil was measured, using the method described in Volz et al. with a duration of 53 s [20].

Calculation of relaxation times:

T1 relaxation times: Quantitative T1 values were calculated in ms according to the variable flip angle method [21]. T1 values were corrected for B1 inhomogeneities and for effects of insufficient spoiling of the transverse magnetization [20].

T2 relaxation times: Quantitative T2 values were calculated in ms via pixel-wise exponential fitting.

T2* relaxation times: Quantitative T2* values were calculated in ms via pixel-wise exponential fitting.

T2′ relaxation times: T2’ was calculated from: 1/T2′ = 1/T2* − 1/T2.

The relative T1 difference pre-to-post GBCA was calculated from: T1rel = (T1 pre-GBCA − T1 post-GBCA)/T1 pre-GBCA.

The assignment of local relaxation times to different biopsy samples was performed with the Brainlab iPlan system (BrainLab iPlan 1.0, Munich, Germany).

### 2.3. Surgical Procedure, Histopathology and MRI Matching

**Figure 1:**
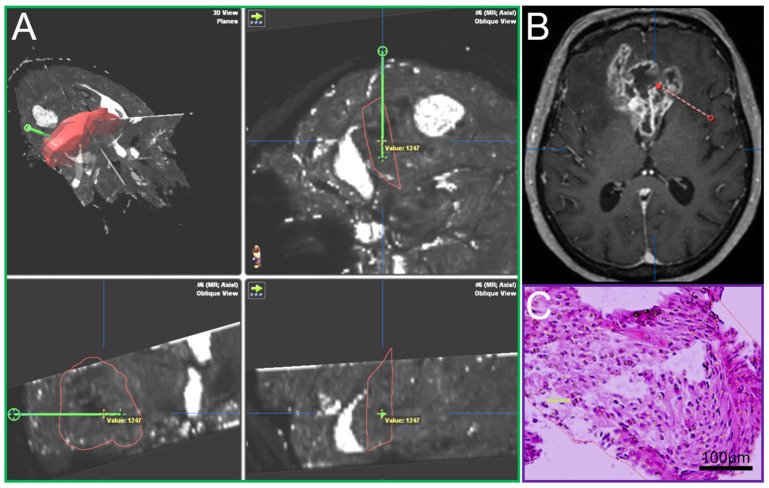
(**A**) T2′ maps with trajectory (green, iPlan BrainLab), (**B**) Conventional MRI, contrast-enhanced T1, axial plane, with trajectory (red), (**C**) Hematoxylin-eosin stain of a biopsy sample.

A Leksell (Leksell^®^ Coordinate Frame G, Elekta Instruments, Stockholm, Sweden) stereotactic frame was attached to the patients’ head prior to burr hole surgery and fixed with four screws (two frontal and two occipital). A CT scan was performed and fused to the MRI. The trajectory was calculated using the BrainLab iPlan system and the localization of each specimen was documented as it was taken on this preplanned trajectory [15]. Such frame-based biopsies have an accuracy within the range of 1–2 mm [22]. In most cases a standard 12 mm burr hole was performed. In order to avoid brain shift the dura was only opened minimally to accommodate the 2.1 mm guide for the biopsy forceps. The number of specimens taken in a tangential manner from tumor areas showing contrast enhancement in MRI [23] and the location of the trajectory were at the discretion of the neurosurgeon in charge and varied due to clinical aspects including the size, the configuration and the location (e.g., eloquent area) of the lesion. The primary aim of the procedure was to confirm the tumor diagnosis employing histology, immunohistochemistry and molecular pathology. An important aspect was the feedback of the neuropathologist’s intra-operative smear-pathology assessment. Up to 23 tissue samples with a volume of 1 mm^3^ in 1 mm steps along the biopsy trajectory were obtained, fixed in 4% paraformaldehyde and paraffin embedded. Sections with a thickness of 3 µm were cut on a Leica SM 2000R microtome (Leica Biosystems, Wetzlar, Germany), mounted on microscope slides (SuperFrost Plus, Thermo Scientific, Waltham, MA, USA) and subjected to HE staining. The O6-methylguanine-DNA methyltransferase (*MGMT*) promoter methylation status was determined by methylation-specific polymerase chain reaction [24]. Immunohistochemistry against isocitrate dehydrogenase (IDH)1_R132H (mouse monoclonal, clone DIA-H09, concentration 1:50, Dianova, Hamburg, Germany), lactate dehydrogenase A (LDHA, Clone C4B5 #3582S, Cell Signaling Technology, Danvers, MA, USA), carbonic anhydrase IX (CAIX, #NB100-417, Novus Biologicals, Littleton, CO, USA) and Ki67 (Clone MIB-1, Dako, Glostrup, Denmark) was performed according to standardized protocols using a Leica BOND-III stainer. The analysis, including cell and vessel counts and the assessment of necrosis on the basis of HE stained sections, was performed by blinded examiners on an Olympus BX41 microscope (Olympus, Tokio, Japan). Pictures were taken with an Olympus DP72 camera and analySIS software provided by Olympus. Cell and vessel density was calculated as the number of cells and vessels per mm^2^. The amount of necrosis (%) was assessed by estimating necrotic areas in relation to the whole tissue area on HE stained slides. The amount of CAIX and LDHA positive areas was determined by estimation of positive stained area in relation to the whole tissue area. Cells stained positive for Ki67 were recorded as percentages of total cellularity. For each sample, it was documented whether vascular proliferates were present or absent. Each tissue probe was correlated with its specific location on the trajectory and the resulting defined position in the MRI data using the BrainLab iPlan system (Figure 1). By doing so, individual quantitative relaxation times were assigned to each biopsy sample.

### 2.4. Statistical Analysis

Data analysis was carried out with SPSS Statistics 26 (IBM, Armonk, NY, USA) and GraphPad Prism 9.1.1 (GraphPad Software, La Jolla, CA, USA). Welch’s t-test was applied for the comparison of relaxation times between two groups [25], i.e., between non-tumor and tumor tissue and between specimens showing vascular proliferates versus specimens without. Spearman rank correlation was used for the estimation of a monotonic relationship between paired data, i.e., between qMRI parameters and histopathological features, as well as between different histopathological variables.

## 3. Results

### 3.1. Quantitative Relaxation Times in Samples Containing Tumor Cells and Vascular Proliferates, Respectively

**Figure 2:**
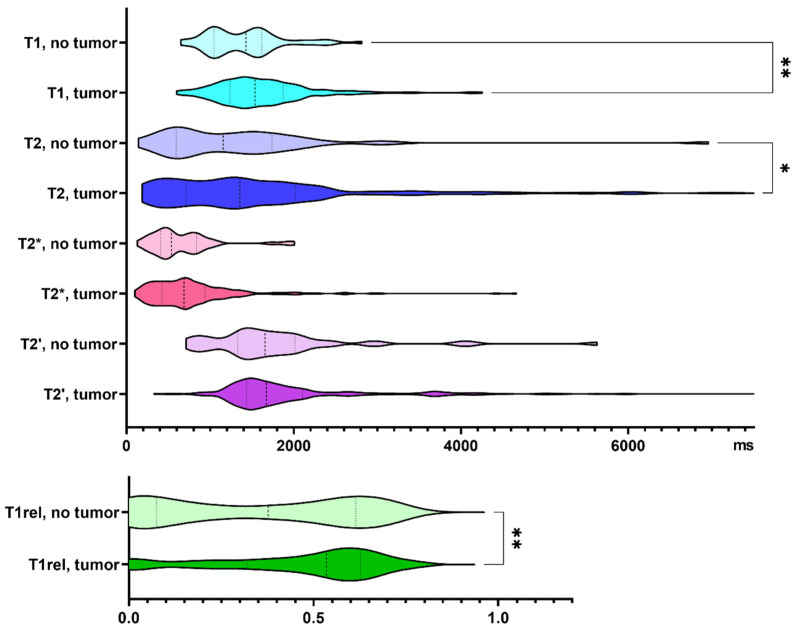
Quantitative relaxation times T1, T2, T2*, T2′ and T1rel in areas without and with tumor cells. The frequency distribution of the data is illustrated by violin plots. The dashed line indicates the median, the dotted lines indicate the 25 and 75 quartiles. * *p* < 0.05, ** *p* < 0.01.

Three hundred and twenty-one biopsy samples from 25 patients with *IDH* wild-type glioma were analyzed. Seventeen percent of the biopsy samples did not contain tumor cells according to neuropathological assessment. Specimens containing tumor cells demonstrated increased T1 and T2 relaxation times (*p* = 0.005, and *p* = 0.013, respectively). T1rel, too, was prolonged in the presence of glioma cells (*p* = 0.009, Figure 2).

**Figure 3:**
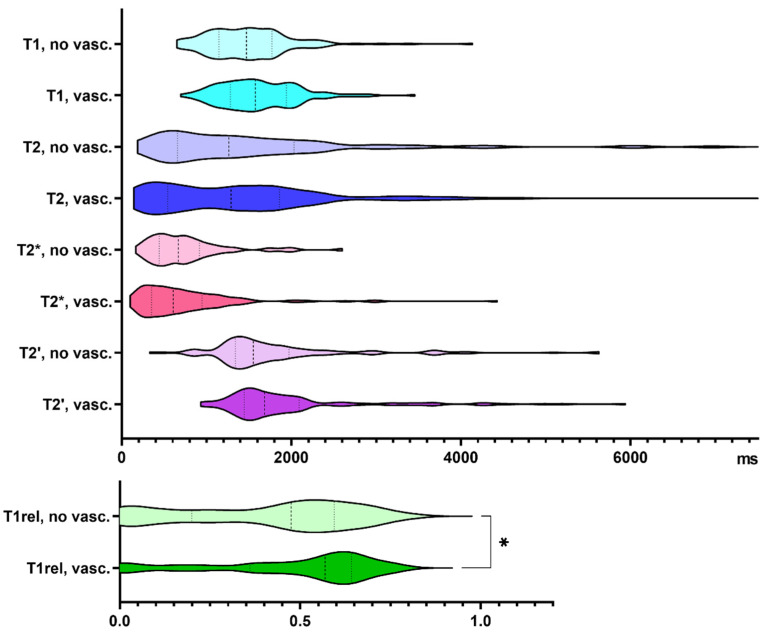
Quantitative relaxation times T1, T2, T2*, T2′ and T1rel in areas without (no vasc.) and with vascular proliferates (vasc.). The frequency distribution of the data is illustrated by violin plots. The dashed line indicates the median, the dotted lines indicate the 25 and 75 quartiles. * *p* < 0.05.

48% of our biopsy samples showed vascular proliferates. Compared to samples without vascular proliferates, T1rel values were significantly prolonged in biopsies with neovascularization (*p* = 0.033, Figure 3). Supplementary Table S1 summarizes the findings exclusively in glioblastoma specimens.

### 3.2. Association of qMRI and Histopathology

Table 3 summarizes correlations between qMRI and histopathological parameters. T1, T2, T2* and T2′ relaxation times were inversely correlated with the vessel density. A weak correlation was found between T1rel and cell density, as well as the Ki67 labeling index, a marker of cell proliferation. The amount of necrosis and the percentage of CAIX-positive cells correlated negatively with T1rel.

### 3.3. Associations between Histopathological Parameters

Correlations between histopathological characteristics are listed in Table 4. The cell density was positively associated with the vessel density and with Ki67 staining, negatively with the proportion of necrosis. The enzyme LDHA catalyzes the conversion of pyruvate into lactate, thus playing a crucial role in glycolysis. Enhanced LDHA expression, as observed in *IDH* wild-type glioma [26], in rapidly growing cells and under hypoxic conditions [27], promotes angiogenesis [28]. In the biopsy samples, immunopositivity for LDHA increased with cell density, the amount of necrosis and CAIX expression. CAIX catalyzes the reversible hydration of carbon dioxide and is also known to be overexpressed in glioblastoma and induced by hypoxia [29]. Consistent with its function in the adaptation of cells to hypoxia, CAIX levels correlated with necrosis and LDHA.

In the presence of tumor cells, cell and vessel densities and the percentages of cells staining positive for CAIX, LDHA and Ki67 exceeded the numbers that were detected in specimens without tumor cells (*p* < 0.001, *p* = 0.006, *p* = 0.003, *p* = 0.002, and *p* < 0.001, respectively). In samples with vascular proliferates, cell density, LDHA and Ki67 expression were higher than in samples without (*p* < 0.001, *p* = 0.035, and *p* = 0.002, respectively).

## 4. Discussion

In the present study, 25 patients with *IDH* wild-type glioma underwent quantitative MR relaxometry and subsequently stereotactic tumor biopsy. Up to 23 tissue samples per patient, 321 in total, were matched to the respective qMRI data. Relaxation times and histopathologic parameters could thus be determined at identical locations.

Quantitative MRI values reflect physical tissue properties, thus indicating physiological and pathological changes of the brain microstructure [3]. T1 values increase both with the intracellular water content (e.g., in the case of cytotoxic edema) and the extracellular water content (e.g., in vasogenic edema and upon loss of solid mass, for example as a consequence of demyelination) [30]. T2 values depend likewise on tissue water environments and vary among different brain structures. They increase with the free water fraction, but are reduced by myelin-bound water [31]. Iron, for example in ferritin, hemoglobin and hemosiderin, shortens T1 and T2 relaxation times [32]. Usually, various processes simultaneously affect T1 and T2. Myelin, for example, is characterized by a high lipid content, water is trapped within lipid bilayers and oligodendrocytes contain iron. Glioma tissue comprises, beside tumor cells, non-tumor cells such as microglia, other immune cells, neurons, astrocytes, endothelial cells, fibroblasts and pericytes, numerous soluble factors and components of the extracellular matrix.

In previous studies, undifferentiated, densely packed neuroblastoma cells with a high nuclear-cytoplasmic ratio and a small extracellular compartment involved higher T1 values [33]. The longitudinal relaxation rate R1 = 1/T1 was also found to be lower in glioblastoma recurrence than in the peritumoral brain zone or the contralateral hemisphere [34]. Early reports on biopsies of primary or secondary brain tumors described increased T1 relaxation times as compared with peritumoral tissue which was partly due to an increased water content in tumor samples [35,36,37].

In accordance, we observed a T1-prolongation in samples containing tumor cells, and histopathological evidence confirmed our assumption of blood brain barrier (BBB) disruption and tumor infiltration beyond the area displaying contrast enhancement in conventional MRI [9]. Another T1-prolonging factor, suggested by the correlation with LDHA expression, might be local hypoxia. Acute ischemia results in a prompt prolongation of T1 and reductions of T2* and T2 [38]. The latter have been attributed to a local increase in deoxyhemoglobin (DeoxyHb) levels. DeoxyHb is paramagnetic, and its presence yields signal reductions in T2-, T2*- and T2′-weighted images [39] with T2′ providing mainly information about local DeoxyHb concentrations. In the present study, we did not observe any association of T2′ and the amount of necrosis, the expression of LDHA and CAIX. In contrast, Klaassen et al. found a positive correlation of R2*, i.e., 1/T2*, with the expression of hypoxia-inducible factor 1-alpha (HIF-1α) in pancreatic ductal adenocarcinoma [14].

However, LDHA and CAIX are indirect markers of hypoxia in tumors whereas T2′ is a marker of DeoxyHb. We observed that lower T2′ values, indicating higher levels of DeoxyHb, were accompanied by higher vessel densities. This might indicate that tumor tissue in these samples had a high oxygen consumption. In line with this hypothesis, we found that a high vessel density in biopsy samples correlated with a high cell density and a high Ki67 index. Deep tumor hypoxia, in contrast, is expected in necrotic tumor areas that are clearly undersupplied with tumor vessels. Therefore, T2′ seems to represent an in vivo marker for deoxygenated tumor vessels and not for hypoxia per se.

Glioblastomas are characterized by abundant vascularization and leaking microvessels with abnormal endothelial wall, pericyte coverage and basement membrane structure, resulting in the loss of BBB integrity [40]. The parameter T1rel represents the leakage of GBCA through the BBB, because T1 is only shortened when GBCA interacts with the tissue outside the vessels. Therefore, this value does not necessarily correspond to vessel density, but mainly represents the leakiness of tumor vessels. Indeed, samples with tumor neovascularization had higher T1rel values, but not samples with just high vessel density. Furthermore, T1rel correlated negatively with the extent of necrosis and immunopositivity for CAIX, and positively with the cell density and the Ki67 index. The negative correlation is due to the absence of enhancement in necrotic tissue. The positive correlation of T1rel with cell density and proliferation is in line with the findings of Barajas et al. [41]. This association may be explained by a higher expression of vascular endothelial growth factor (VEGF) in samples with a high number of glioblastoma cells [42]. One major player in increasing vessel permeability is VEGF, which is highly expressed in glioblastoma cells. However, VEGF also contributes significantly to an increase in vessel density. Therefore, our results might indicate the existence of other mechanisms that increase BBB damage in the presence of proliferating glioblastoma cells. Another relevant cause for a BBB disruption is the co-option of preexisting vessels by glioma cells [43]. It has been shown that tumor cells populate the perivascular space of preexisting vessels and displace astrocytic endfeet from endothelial or vascular smooth muscle cells. This leads to an abnormal BBB permeability [44]. Vessel co-opting glioma cells benefit from oxygen and nutrient supply; the microenvironment of this specific vascular niche again stimulates the proliferation of the tumor cells. Therefore, angiogenesis is probably only one of many factors that contribute to the BBB damage in glioblastomas. In line with this, our results and previous experimental evidence found a negative association of microvessel density and T1 relaxation time [45]. This negative correlation indicates that the BBB integrity is at least partially maintained in these tumor vessels, because otherwise tissue water should be increased, which in turn would increase the T1 and T2 relaxation times.

The T2 relaxation time has been shown to be a sensitive marker indicating brain pathologies such as multiple sclerosis [46], trauma [47] or epilepsy [48]. The longer T2 values in biopsy samples with tumor cells are in line with this concept. However, an increase in T2 seems to be little specific. We therefore assume that quantitative T2 mapping may provide added value especially in longitudinal studies [12].

A limitation of our study is the potential misregistration between MRI and biopsy due to a brain shift caused by the opening of the dura mater. We therefore cannot rule out some mismatch but tried to minimize this source of error by the accurate use of the intraoperative neuronavigational system (BrainLab iPlan). It should be noted that Brainlab iPlan transforms data into an internal scaling system with a limited range of numbers. Therefore, real quantitative values were lost, and only relative values could be documented in the target regions. There was also a relevant discrepancy between the spatial resolution of an image voxel (T1: 1 mm^3^, T2 and T2*: 3.125 mm^3^) and the size of the neurosurgical sample (1 mm^3^). Another limitation is the rather small number of patients. Nevertheless, this study showed that such investigations, which contribute to the understanding of (patho)physiological processes, are feasible. Furthermore, the biopsies could have been processed more extensively, e.g., for DNA methylation analysis. As the generation of qMRI maps requires the acquisition of multiple image series, these maps are more susceptible to motion artifacts. Examination times are longer than those typically required in conventional MRI and may not be suitable for investigating severely ill or less cooperative patients. This problem will probably be overcome by further technical and software development. Next, reference ranges must be defined for the individual parameters.

The interpretation of qMRI maps which are given in physical units is more precise than the analysis of conventional MRI data, where signal values are arbitrarily scaled, and image contrast often results from a mixture of several parameters. Quantitative data are not only objective, but also sensitively detect pathologies and allow further analysis by means of artificial intelligence. For these reasons, information derived from multiparametric imaging could help to improve the extent of tumor resection, the target volume definition for radiotherapy and the response assessment.

## 5. Conclusions

MRI is a powerful tool for the evaluation of intracranial masses and guides neurosurgeons when performing biopsies for the diagnosis of gliomas. The histopathological validation of imaging findings allows for (i) the verification of concepts underlying the use of imaging modalities and (ii) an advanced interpretation of pathologically defined structural abnormalities. Taken together, in the present study, we found an increase in T1 and T2 relaxation times, as well as in T1rel, in the presence of tumor cells. Vascular proliferates, but not vessel densities, were associated with a stronger reduction of T1 post-GBCA. Contrary to what might have been expected, there was no significant correlation between T2′ and two indirect indicators of hypoxic conditions, LDHA and CAIX. However, a relationship between T2′ relaxation times and the vessel density was observed.

## Figures and Tables

**Table 1:** Patient and tumor characteristics. *IDH*, isocitrate dehydrogenase. *MGMT*, O6-methylguanine-DNA methyltransferase.

**Sex [n]**	Female	9
	Male	16
**Age [years]**	Median	65
	Range	27–88
**Diagnosis [n]**	*IDH* wild-type glioblastoma	23
	*IDH* wild-type astrocytoma, WHO grade III	1
	*IDH* wild-type astrocytoma, WHO grade II	1
***MGMT* Promoter [n]**	Unmethylated	10
	Methylated	9
	*MGMT* status not available/inconclusive	6
**Samples Per Patient [n]**	Median	13
	Range	4–23
**Survival [Days]**	Median	295
	Range	3–930

**Table 2:** Quantitative MRI parameters. FLASH-EPI: 3D gradient echo (GE) sequence with a FLASH (fast low-angle shot)—EPI (echo planar imaging) hybrid readout [17] and fat-insensitive excitation pulses [18]. GBCA: application of standardized intravenous contrast agent injection (0.05 mmol/kg gadobutrol; Gadovist, Bayer AG, Leverkusen, Germany).

Relaxation Time	Sequence	Field of View	Matrix	Repetition Time TR [ms]	Echo Time(s) TE [ms]	Flip Angle [°]	Voxel Size [mm^3^]	Bandwidth Hz/Pixel	Acquisition Time [min]
T1 pre GBCA	3D FLASH-EPI	256 × 224 × 160 mm^3^	256 × 224 × 160	16.4	6.7	4/24	1 × 1 × 1	222	9:48
T2	2D Turbo Spin Echo	240 × 180 mm^2^	192 × 144	4670	16, 64, 96, 128, 176	90/180	1.25 × 1.25 × 2	100	5 × 1:12
T2*	2D Multi-Echo Gradient Echo	240 × 180 mm^2^	192 × 144	1500	10, 16, 22, 28, 34, 40, 46, 52	30	1.25 × 1.25 × 2	299	6:36
T1 post GBCA	3D FLASH-EPI	256 × 224 × 160 mm^3^	256 × 224 × 160	16.4	6.7	4/24	1 × 1 × 1	222	9:48

**Table 3:** Correlations of qMRI parameters and neuropathological features. Significant correlations are highlighted in grey. CAIX, carbonic anhydrase IX, LDHA, lactate dehydrogenase A, r_S_ = Spearman correlation coefficient, *p* = *p*-value. For glioblastoma samples only, see Supplementary Table S2.

	Cell Density [Cells/mm^2^]		Vessel Density [Vessels/mm^2^]		Necrosis [%]		CAIX [%]		LDHA [%]		Ki67 [%]	
	r_S_	*p*	r_S_	*p*	r_S_	*p*	r_S_	*p*	r_S_	*p*	r_S_	*p*
T1	−0.010	0.853	−0.248	<0.001	0.065	0.245	0.071	0.263	0.249	<0.001	0.001	0.988
T1rel	0.116	0.050	−0.050	0.448	−0.195	0.001	−0.191	0.004	0.072	0.276	0.140	0.038
T2	0.017	0.762	−0.200	0.002	−0.093	0.107	−0.029	0.661	0.151	0.019	0.070	0.297
T2*	0.014	0.803	−0.235	<0.001	−0.093	0.110	−0.017	0.797	0.212	0.001	0.014	0.833
T2′	0.009	0.870	−0.264	<0.001	0.002	0.968	−0.125	0.057	0.066	0.310	0.078	0.243

**Table 4:** Correlations between histopathological features. Significant correlations are highlighted in grey. r_S_ = Spearman correlation coefficient, *p* = *p*-value. For glioblastoma samples only, see Supplementary Table S3.

	Cell Density [Cells/mm^2^]		Vessel Density [Vessels/mm^2^]		Necrosis [%]		CAIX [%]		LDHA [%]	
	r_S_	*p*	r_S_	*p*	r_S_	*p*	r_S_	*p*	r_S_	*p*
vessel density [vessels/mm^2^]	0.272	<0.001								
necrosis [%]	−0.314	<0.001	−0.161	0.012						
CAIX [%]	0.053	0.414	−0.082	0.200	0.536	<0.001				
LDHA [%]	0.204	0.001	−0.072	0.264	0.443	<0.001	0.583	<0.001		
Ki67 [%]	0.340	<0.001	0.142	0.030	−0.145	0.029	−0.081	0.221	0.042	0.525

## Data Availability

The data presented in this study are available on request from the corresponding author.

## Supplementary Materials

The following are available online at https://www.mdpi.com/article/10.3390/cancers13164060/s1, Figure S1: Schematical description of the derivation of quantitative T2, T2* and T2’ maps, Table S1: Quantitative relaxation times depending on the presence of tumor cells or vascular proliferates in all *IDH* wild-type gliomas and glioblastomas only, Table S2: Correlations of qMRI parameters and neuropathological features in glioblastoma samples, Table S3: Correlations between histopathological features in glioblastoma samples.
