# Supplementary material for: Matching Quantitative MRI Parameters with Histological Features of Treatment-Naïve IDH Wild-Type Glioma
Source: Cancers (Basel). 2021 Aug 12;13(16):4060. doi: 10.3390/cancers13164060 (PMC8392045; doi:10.3390/cancers13164060)
Supplement: Supplementary file 1 [file cancers-13-04060-s001.zip › cancers-1277356-supplementary.pdf]

Supplementary Figure S1

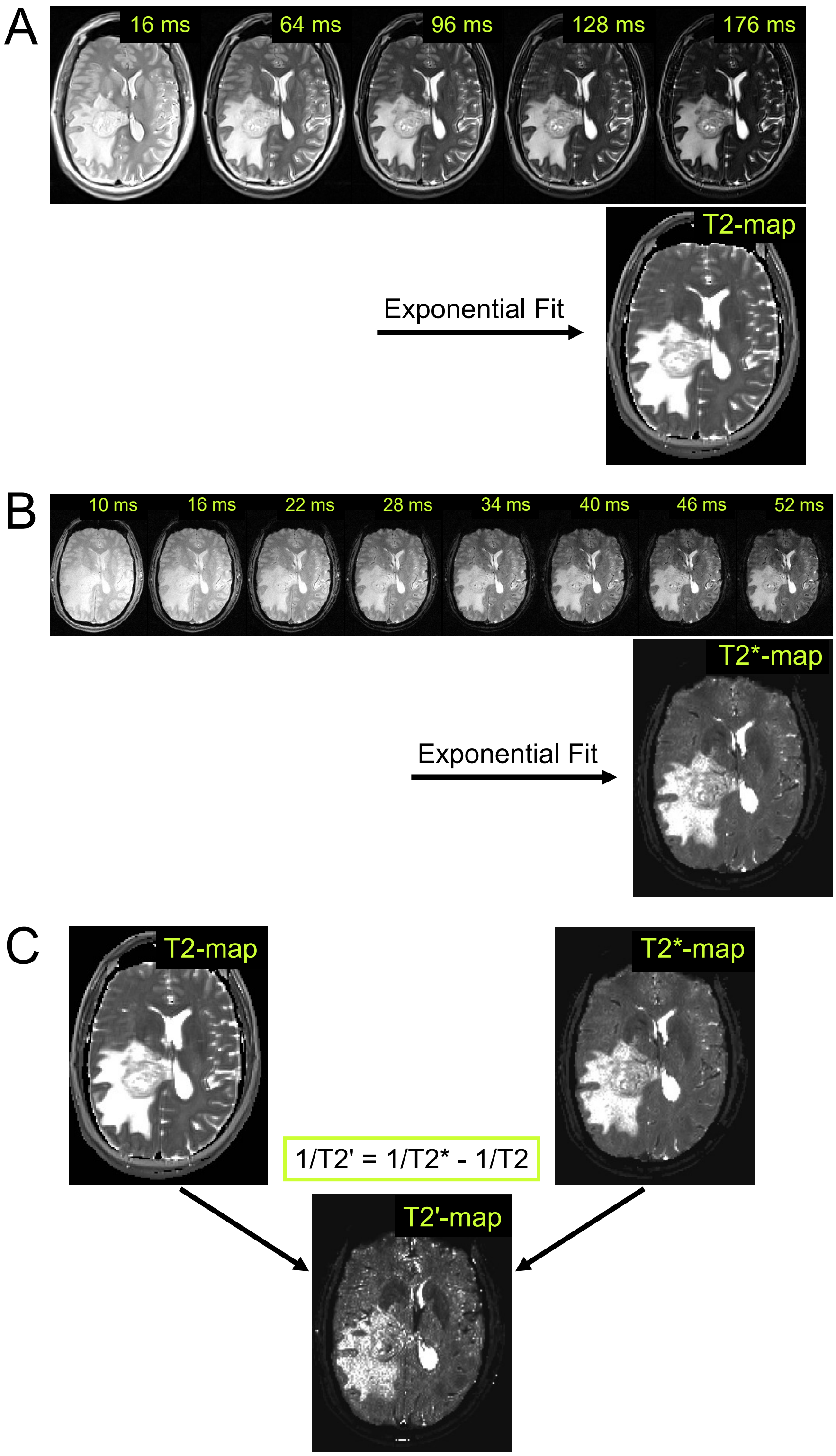

Supplementary Table S1

Analysis of all 25 *IDH*-wildtpye gliomas (n = 25) and those gliomas that histologically had been classified as glioblastomas (n = 23). Groups of non-tumor versus tumor samples and groups of samples with vascular proliferates (vasc.) versus samples without (no vasc.) were compared using the Welch's t-test. Note that biopsy specimens from the two patients with astrocytoma did not show vascular proliferates. N.s.: not significant.

**All gliomas (25)**	**parameter**	**group**	**mean**	**median**	***p***
	T1	no tumor	1408	1429	0.005
		tumor	1622	1537	
	T2	no tumor	1318	1156	0.013
		tumor	1802	1356	
	T2*	no tumor	665	537	n.s.
		tumor	796	687	
	T2‘	no tumor	1816	1657	n.s.
		tumor	2004	1672	
	T1rel	no tumor	0.305	0.377	0.009
		tumor	0.445	0.534	
**Glioblastomas (23)**	**parameter**	**group**	**mean**	**median**	***p***
	T1	no tumor	1360	1231	0.001
		tumor	1598	1510	
	T2	no tumor	1337	1153	0.029
		tumor	1837	1367	
	T2*	no tumor	673	537	n.s.
		tumor	813	700	
	T2‘	no tumor	1784	1685	n.s.
		tumor	1998	1685	
	T1rel	no tumor	0.279	0.309	0.002
		tumor	0.444	0.534	
**All gliomas (25)**	**parameter**	**group**	**mean**	**median**	***p***
	T1	no vasc.	1502	1470	n.s. (0.053)
		vasc.	1622	1576	
	T2	no vasc.	1765	1263	n.s. (0.080)
		vasc.	1422	1288	
	T2*	no vasc.	756	668	n.s.
		vasc.	755	608	
	T2‘	no vasc.	1823	1551	n.s.
		vasc.	1977	1684	
	T1rel	no vasc.	0.385	0.475	0.033
		vasc.	0.451	0.569	
**Glioblastomas (23)**	**parameter**	**group**	**mean**	**median**	***p***
	T1	no vasc.	1467	1389	0.018
		vasc.	1622	1576	
	T2	no vasc.	1877	1359	0.033
		vasc.	1422	1288	
	T2*	no vasc.	794	720	n.s.
		vasc.	755	608	
	T2‘	no vasc.	1885	1624	n.s.
		vasc.	1977	1684	
	T1rel	no vasc.	0.385	0.475	0.033
		vasc.	0.451	0.569	

Supplementary Table S2: Correlations of qMRI parameters and neuropathological features in glioblastoma samples. Significant correlations are highlighted in grey. r_S = Spearman correlation coefficient, *p* = p-value.

	**cell density [cells/mm²]**		**vessel density [vessels/mm²]**		**necrosis [%]**		**CAIX [%]**		**LDHA [%]**		**Ki67 [%]**	
	r_S	*p*	r_S	*p*	r_S	*p*	r_S	*p*	r_S	*p*	r_S	*p*
**T1**	0.001	0.990	-0.234	<0.001	0.081	0.166	0.084	0.193	0.240	<0.001	0.007	0.911
**T1rel**	0.123	0.041	-0.037	0.578	-0.193	0.002	-0.201	0.002	0.070	0.292	0.144	0.036
**T2**	-0.009	0.874	-0.190	0.003	-0.123	0.039	-0.025	0.698	0.143	0.028	0.073	0.276
**T2***	-0.010	0.859	-0.230	<0.001	-0.116	0.053	-0.017	0.797	0.208	0.001	0.015	0.818
**T2'**	-0.011	0.854	-0.262	<0.001	-0.044	0.463	-0.118	0.074	0.064	0.334	0.079	0.240

Supplementary Table S3: Correlations between histopathological features in glioblastoma samples. Significant correlations are highlighted in grey. r_S = Spearman correlation coefficient, *p* = p-value.

	**cell density [cells/mm²]**		**vessel density [vessels/mm²]**		**necrosis [%]**		**CAIX [%]**		**LDHA [%]**	
	r_S	*p*	r_S	*p*	r_S	*p*	r_S	*p*	r_S	*p*
**vessel density [vessels/mm²]**	0.278	<0.001								
**necrosis [%]**	-0.357	<0.001	-0.157	0.015						
**CAIX [%]**	0.033	0.611	-0.094	0.150	0.549	<0.001				
**LDHA [%]**	0.202	0.002	-0.052	0.421	0.442	<0.001	0.591	<0.001		
**Ki67 [%]**	0.325	<0.001	0.137	0.040	-0.156	0.021	-0.106	0.113	0.036	0.594
